# Intranasal Dexmedetomidine for the Treatment of Pre-operative Anxiety and Insomnia: A Prospective, Randomized, Controlled, and Clinical Trial

**DOI:** 10.3389/fpsyt.2022.816893

**Published:** 2022-05-30

**Authors:** Wen Zeng, Li Chen, Xin Liu, Xujiang Deng, Kuan Huang, Maolin Zhong, Shubao Zhou, Lifang Zhan, Yulu Jiang, Weidong Liang

**Affiliations:** ^1^The First School of Clinical Medicine, Gannan Medical University, Ganzhou, China; ^2^Department of Anaesthesiology, First Affiliated Hospital of Gannan Medical University, Ganzhou, China; ^3^Department of Obstetrics and Gynecology, Luhe Hospital, Yingkou, China

**Keywords:** intranasal dexmedetomidine, pre-operative anxiety and insomnia, Narcotrend index, insomnia severity index, dexmedetomidine

## Abstract

**Background and Objective:**

Several patients with pre-operative anxiety and insomnia refuse to take sleeping pills because of the side effects of sleeping pills. This study aimed to evaluate the applicability of intranasal dexmedetomidine (DEX) in the treatment of pre-operative anxiety and insomnia.

**Methods:**

A total of 72 patients with insomnia and anxiety were randomly divided into two groups of intranasal DEX (n = 36) and intranasal normal saline (NS, n = 36). The primary outcomes included patients' time to fall asleep, total sleep time, insomnia severity index (ISI) after treatment, and satisfaction with the treatment effect. The secondary outcomes were mean arterial pressure (MAP), oxygen saturation (SPO_2_), heart rate (HR), Narcotrend index (NI) in the first 2 h of treatment, and the incidence of adverse events within 12 h after treatment.

**Results:**

The time to fall asleep (22.08 ± 3.95 min) and total sleep time (400.06 ± 28.84 min) in the DEX group were significantly different from those in the NS group [time to fall asleep, 89.31 ± 54.56 min; total sleep time (295.19 ± 73.51 min; *P* < 0.001)]. ISI after treatment in the DEX group was lower than that in the NS group (*P* < 0.001). Satisfaction with the treatment effect was better in the DEX group than that in the NS group (*P* < 0.001). The general vital signs in the two groups were stable during the treatment. The drowsiness rate in the NS group was higher than that in the DEX group (*P* < 0.001).

**Conclusion:**

Intranasal DEX can significantly improve pre-operative anxiety and insomnia.

**Clinical Trial Registration:**

This study was registered on Chinese Clinical Trial Registry (http://www.chictr.org.cn/searchproj.aspx, ChiCTR2100044747).

## Introduction

Insomnia refers to a subjective experience of patients who are not satisfied with the time or quality of sleep, influencing their social functions. It is mainly characterized by dissatisfaction with sleep quantity or quality, associated with difficulty falling asleep, frequent nighttime awakenings with difficulty returning to sleep, and/or awakening earlier in the morning than desired (1–3). A recent study showed that the incidence of insomnia among the general population in China is 12.1–18.5% (4). Multiple factors are associated with sleep disorders in hospitalized patients, including ambient environmental noise, the underlying acute illness, pain, anxiety, depression, delirium, etc. (5). The incidence of pre-operative insomnia is 37–38.2% (6, 7). Such a high incidence requires clinicians' additional attention to provide more efficacious treatments for patients with insomnia.

A growing body of evidence demonstrated that there is an association between pre-operative insomnia and short-term and long-term adverse post-operative consequences. Insomnia can damage immune function (8), and it is related to a high blood pressure (BP) and other cardiovascular risk factors (9, 10), a poor blood sugar control (11, 12), cognitive impairment (13, 14), and mental health (15). In addition, pre-operative insomnia causes patients' poor medical experience, while increases the incidence of disease complications (16, 17).

Improving patients' pre-operative sleep quality may accompany by positive consequences and improve safety in the perioperative period. To date, benzodiazepine and non-benzodiazepine hypnotics were commonly used to improve patients' pre-operative sleep quality, which were mainly administered orally and intravenously. Oral administration of sleeping pills may improve short-term sleep outcomes in adults with insomnia, whereas sleeping pills may cause cognitive and behavioral abnormalities (18). Intravenous injection is often accompanied by pain, and the drug dose is difficult to titrate (19). Additionally, some patients were worried about the addiction and side effects of sleeping pills, and resisted taking sleeping pills may assist them to sleep easier. Different from other sedatives acting on GABA, dexmedetomidine (DEX) is a highly selective α2-adrenergic receptor (α2-AR) agonist that is associated with sedative and analgesic sparing effects, as well as reduced delirium and agitation. Intranasal DEX eliminates patients' need to an open vein on the night before surgery. DEX is absorbed by the central nervous system through the capillaries of the nasal cavity and promotion of endogenous sleep pathways, leading to produce sedative and hypnotic effects. Although no previous study has investigated the effectiveness of intranasal DEX for adult patients with insomnia, there is a report of a case of successful long-term home use of intranasal DEX for insomnia in pediatric palliative care of a 10-year-old women with dystrophic epidermolysis bullosa and severe sleep disorders, where treatment resulted in an increase in sleep duration from 2 to 3 consecutive hours to 6–8 consecutive hours (20). Studies suggested that DEX-induced deep sedation mimics stages 2 and 3 of non-rapid eye movement (NREM) sleep (21, 22). In particular, it does not cause respiratory depression (23).

Hence, the present study aimed to evaluate the feasibility of administration of intranasal DEX in the treatment of pre-operative anxiety and insomnia.

## Materials and Methods

### Ethics Approval

The present study was approved by the Scientific Research Ethics Committee of the First Affiliated Hospital of Gannan Medical University (LLSC-2020102701), and registered on Chinese Clinical Trial Registry (ChiCTR2100044747). The study protocol was conducted in accordance with the Declaration of Helsinki. All participants were informed about the objectives of the study by investigators. All participants provided written informed consent before enrollment.

### Participants

The research was conducted in the First Affiliated Hospital of Gannan Medical University (Ganzhou, China) between April and October 2021. The following inclusion criteria were used: 1. Patients with American Association of Anesthesiologists (ASA) grade I II; 2. Patients with Hamilton Anxiety Rating Scale (HAMA) score ≥ 7 points; 3. Patients with insomnia severity index (ISI) score ≤7 points (one week before hospitalization), and ISI >7 points (after hospitalization); 4. Patients with blood oxygen saturation > 95%; 5. Patients who aged 18-60 years old, regardless of gender; 6. Patients with body mass index (BMI) <30 kg/m^2^; 7. Patients who participated in completing the questionnaire; 8. Patients with insomnia and were unwilling to use benzodiazepines.

The exclusion criteria were as follows: 1. Patients who were diagnosed or suspected of having sleep apnea syndrome; 2. Patients with alcohol or drug addiction; 3. Patients who have used antidepressants or psychiatric drugs 1 week before enrollment; 4. Patients who were allergic to DEX or other drugs used in this study; 5. Patients with abnormal liver or kidney function; 6. Pregnant or breastfeeding women.

### Assessment of Insomnia and Anxiety

Insomnia is defined as chronic dissatisfaction with sleep quantity or quality. Therefore, self-assessment of insomnia severity is important for more effective treatment of insomnia. In insomnia studies, the ISI and the Pittsburgh Sleep Quality Index (PSQI) are recommended measures for overall sleep and insomnia symptoms (24). The majority of ISI-related studies concentrated directly on patients' subjective feelings in association with insomnia symptoms. The PSQI was originally designed to assess general sleep quality over a period of 1 month (25). The Chinese version of ISI successfully distinguished insomnia patients from healthy participants with sensitivity and specificity >0.9 (26). A study conducted in Canada, Hong Kong, and Taiwan further supported the structural validity and cross-cultural comparability of ISI (27). Chen et al. (26) evaluated whether ISI and PSQI were effective outcome indicators of cognitive behavioral therapy for insomnia, suggesting that ISI might be a more reliable scoring scale. Therefore, ISI was used to evaluate the severity of insomnia in the present study. There were 7 items, and each item scored 0–4 points, with a total score of 28 points. A score of more than 8 points was considered as a sleep disorder.

The HAMA includes 14 interview items, with a 5-point scale ranging from 0 (non-existent) to 4 (very severe). Higher scores of HAMA indicate more severe symptoms of anxiety. An overall score of 8 points or higher indicates anxiety.

### Narcotrend Index (NI)

The gold standard for sleep stage detection is the sleep multi-channel monitor, however, patients need to be transferred from an adapted ward to a new environment for evaluation. The Observer's Assessment of Alertness/Sedation Scale (OAA/S) can be used to assess patients' sleep status that may interrupt patients' sleep. Narcotrend is an electroencephalogram (EEG) monitor designed to measure the depth of anesthesia. The NI is dimensionless, ranging from 0 to 100. The NI could be divided into six stages [A (awakening) to F (anesthesia with outbreak suppression)] (Supplementary Table S1). The NI has a strong correlation with OAA/S, which was supported by Bauerle et al.'s results (28). The selected index of the experiment is the C-F stage which means that the patient was asleep. If the depth of anesthesia does not reach the target state for more than 30 min, the treatment fails.

### Methods

Patients were randomly allocated to two groups by a computer-generated sequence. Sleep assessment was performed in the participant's ward on the night before surgery. Bedtime was adjusted according to the patient's sleep habits. Normal bedtime was controlled from 21:00 to 23:00. The NI was used to monitor the patient's EEG and depth of sedation at bedtime, and to simultaneously control the patient's mean arterial pressure (MAP), heart rate (HR), and oxygen saturation (SPO_2_). Patients were enrolled in the present study without receiving oxygen through a nasal catheter. Patients administered by the same unsuspecting anesthesiologist through the nasal cavity with a preconfigured drug. At present, there is no relevant study on 95% effective dose of intranasal DEX in adults. According to Li et al.'s study (23), 95% effective dose of intranasal DEX in children was 2.64 μg/kg. Referring to this dose, we carried out a pre-experiment *via* setting up three experimental groups (2.0, 2.5, and 3.0 μg/kg). According to the pre-experiment results, the experimental dose was determined to be 2.5 μg/kg. The dosage was divided evenly between the two sides of the nostrils, in which 0.1 ml was given to each side of the nostrils, and the two sides were alternately given. After each round of administration, gently press on the client's nose and administer the required dose within 5 min. On the next day, the fuzzy number method was utilized to evaluate the degree of satisfaction with the night's sleep. The total score was 10 points, and the final result was 8 points or more, indicating that the patient was satisfied with the sleep. It is noteworthy that final score of 6–8 points indicated that the patient was generally satisfied, and when the final score was <6 points, the patient was not satisfied. Besides, ISI was applied to evaluate the night's sleep. Patients, nurses, and investigators were blinded to the grouping.

Throughout the research process, we collected the following data: sleep onset latency, total sleep time, ISI and HAMA scores before treatment, ISI and satisfaction scores after treatment, MAP, HR, SPO_2_ at 0 min (T_0_), SPO_2_ at 10 min (T_1_), SPO_2_ at 20 min (T_2_), SPO_2_ at 30 min (T_3_), SPO_2_ at 1 h (T_4_), and SPO_2_ at 2 h (T_5_) after treatment.

### Interventional Conditions

Hypotension was defined as a decrease in MAP >30% from baseline (before treatment). BP was controlled within 30% of baseline. If BP is outside the target range, phenylephrine may increase BP, and nitroglycerin may decrease BP. If the HR drops below 40 beats/min, atropine may be used, and isoproterenol may be used when atropine is ineffective. Patients were given nasal cannula when SPO_2_ was below 90%. The anesthesiologist determined the necessity of further medical management.

### Adverse Reactions

Adverse reactions, such as respiratory depression, hypotension, and sinus bradycardia were recorded throughout the treatment period.

### Statistical Analysis

SPSS 21.0 software (IBM, Armonk, NY, USA) and GraphPad Prism 8.0 software (GraphPad Software Inc., San Diego, CA, USA) were used to perform the statistical analysis. The Shapiro–Wilk test was used to assess the distribution of continuous variables that were presented as mean [standard deviation (SD)]; categorical variables were expressed as percentage. Normally distributed data were analyzed by one-way analysis of variance (ANOVA) or paired *t*-test to compare differences between groups; abnormally distributed data were analyzed using the non-parametric test to compare differences between groups (the Mann–White *U* test for two independent samples). Comparison of categorical variables between the groups was carried out using the χ^2^ test. A two-sided *P* < 0.05 was considered statistically significant.

### Sample Size Calculation

A previous study reported that the incidence of pre-operative insomnia is 37–38.2% (6, 7). They assumed that after intranasal DEX administration, the incidence of pre-operative insomnia decreased from 38.2 to 5%, with the statistical power of 90% and a two-sided significance level of 0.05. Besides, 65 patients were required to detect a statistical significance. They considered a loss to follow-up rate of ~10%. A total of 72 patients were enrolled in the present study.

## Results

**Figure 1:**
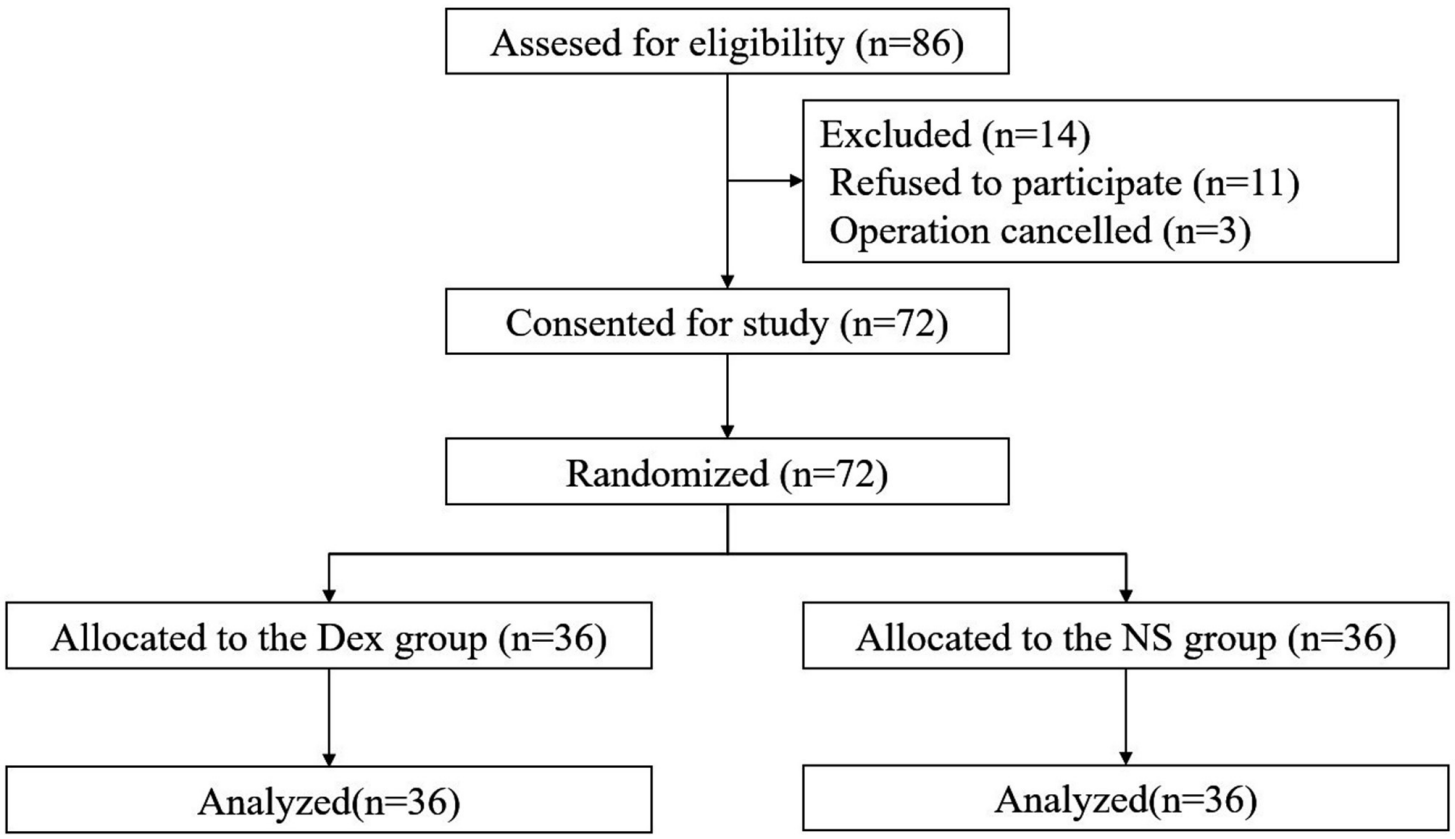
Study flow diagram of participants in the randomized trial. Dex, Intranasal dexmedetomidine group; NS, Intranasal normal saline group.

Among 86 patients who were enrolled, 11 patients refused participation in the study, and 3 patients were excluded due to inconsistency with the surgical plan. A total of 72 patients were finally included and randomly assigned into the DEX group (*n* = 36) and intranasal normal saline (NS) group (*n* = 36). No patients withdrew from the study. The study flow diagram is shown in Figure 1. Demographic data, ASA grade, standard bodyweight, NI, ISI (before hospitalization), and ISI and HAMA scores before treatment were similar between the two groups (Table 1).

**Table 1:** Characteristics of study population.

**Characteristic**	**Dex (*n* = 36)**	**NS (*n* = 36)**	***P-*value**
Age (year)	38.11 ± 11.47	40.47 ± 11.17	0.539
Sex (males)	15 (41.7%)	14 (38.9%)	0.810
Height (cm)	164.44 ± 6.87	164.78 ± 7.45	0.648
Standard body weight (Kg/m^2^)	57.92 ± 5.46	58.11 ± 5.90	0.866
Body mass index (Kg/m^2^)	22.11 ± 2.09	22.23 ± 2.05	0.416
ASA I/II(No)	15/21	13/23	0.515
Baseline heart rate (beats/min)	74.47 ± 8.77	76.58 ± 10.72	0.306
Baseline mean arterlal pressure (mm Hg)	89.17 ± 6.36	87.25 ± 7.86	0.426
Baseline oxygen saturation	97.75 ± 1.08	97.83 ± 0.94	0.687
Narcotrend index	98.17 ± 0.85	98.17 ± 0.74	0.914
Before hospitalization ISI	4.36 ± 1.42	4.22 ± 1.35	0.981
ISI	13.53 ± 4.14	13.50 ± 4.41	0.860
HAMA	14.11 ± 3.23	13.58 ± 2.59	0.637

*Values expressed as mean ± standard devlation or number (percentage)*.

*No differences were found (P < 0.05)*.

*Dex, Intranasal dexmedetomidine group; NS, Intranasal Normal saline group; ISI, Insomnia severity index; HAMA, Hamilton Anxiety Scale; ASA, American Society of Anesthesiology*.

### Primary Outcome Measures

**Figure 2:**
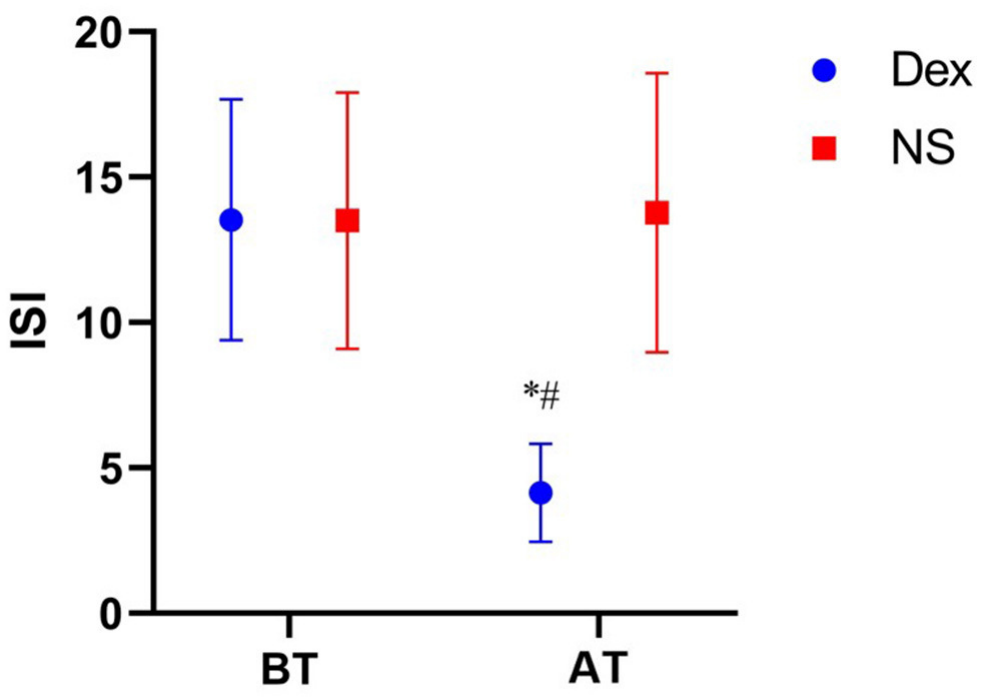
Comparison of ISI scores in two groups at different time points. BT, Before treatment; AT, After treatment; ISI, Insomnia severity index; Dex, Intranasal dexmedetomidine group; NS, Intranasal normal saline group. ISI score comparison of Dex group before and after treatment, *P* < 0.01 vs. *. Comparison of ISI score between Dex group and NS group after treatment, *P* < 0.01 vs. ^#^.

(1) The time to fall asleep (22.08 ± 3.95 min) and total sleep time (400.06 ± 28.84 min) in the DEX group were significantly different from those in the NS group (time to fall asleep, 89.31 ± 54.56 min; total sleep time (295.19 ±7 3.51 min; *P* < 0.001). (2) The overall sleep satisfaction rate on the night before surgery was significantly different between the two groups (*P* < 0.001) (Table 2). (3) The pre-operative ISI scores were similar in the DEX (13.53 ± 4.14) and NS (13.50 ± 4.41) groups (*P* = 0.860). ISI score after treatment in the DEX group (4.14 ± 1.68) was lower than that in the NS group (13.78 ± 4.80) (*P* < 0.001). The ISI score in the DEX group (4.14 ± 1.68, after treatment) was significantly lower than that in the DEX group (13.53 ± 4.14, before treatment; *P* < 0.001), whereas no significant differences were found between before and after treatment in the NS group (*P* = 0.636) (Figure 2).

**Table 2:** Comparison of fall asleep time, total sleep time and satisfaction of patients in two groups ($\bar{x}\pm$ s).

	**Dex**	**NS**	***P***
Fall asleep time	22.08 ± 3.95	89.31 ± 54.56	<0.001
Total sleep time	400.06 ± 28.84	295.19 ± 73.51	<0.001
satisfaction of patients	8.14 ± 0.87	3.50 ± 1.66	<0.001

*Dex, Intranasal dexmedetomidine group; NS, Intranasal Normal saline group*.

### Secondary Outcome Measures

The MAP, HR, SPO_2_, and NI are summarized in Table 3. Figure 3 shows the effects of DEX dose over time on MAP, HR, SPO_2_, and NI. 1. MAP and HR in the DEX group were significantly different from those in the NS group from T_2_ to T_5_ (*P* < 0.05) 0.2. The SPO_2_ after treatment did not significantly differ between the two groups at T_0_, T_1_, T_3_, and T_5_ (*P* > 0.05); at T_2_ and T_4_, the SPO_2_ in the DEX group (97.17 ± 0.91 at T_4_, 97.19 ± 0.95 at T_5_) was significantly lower than that in the NS group (97.67 ± 0.86 at T_4_, 97.61 ± 0.73 at T_5_; *P* < 0.05). However, at T_2_ and T_4_, the SPO_2_ did not fall below 95%, thus, the difference was not clinically significant. 3. At T_2_, T_3_, T_4_, and T_5_ after treatment, the NI in the DEX group (77.00 ± 28.77 at T_2_, 35.08 ± 11.73 at T_3_, 35.31 ± 5.85 at T_4_, and 36.08 ± 5.68 at T_5_) was significantly lower than that in the NS group (98.31 ± 0.67 at T_2_, 94.44 ± 1 3.39 at T_3_, 73.19 ± 2 9.61 at T_4_, and 51.67 ± 26.12 at T_5_; *P* < 0.05).

**Table 3:** Comparison at each time point in two groups ($\bar{x}\pm$ s).

	**T_**0**_**	**T_**1**_**	**T_**2**_**	**T_**3**_**	**T_**4**_**	**T_**5**_**
**MAP**						
Dex	89.17 ± 6.36	83.24 ± 5.98^*^	78.83 ± 6.22^*^^#^	76.59 ± 5.63^*^^#^	74.98 ± 5.41^*^^#^	76.14 ± 5.05^*^^#^
NS	87.29 ± 7.86	85.35 ± 8.67	85.56 ± 9.24	84.31 ± 8.38^&^	81.82 ± 9.01^&^	79.95 ± 9.06^&^
**HR**						
Dex	74.47 ± 8.77	66.75 ± 8.42^*^^#^	60.00 ± 8.52^*^^#^	57.28 ± 7.59^*^^#^	55.86 ± 6.99^*^^#^	56.69 ± 6.24^*^^#^
NS	76.58 ± 10.72	73.53 ± 11.91	71.72 ± 12.27^&^	69.92 ± 13.80^&^	65.50 ± 11.59^&^	63.08 ± 9.44^&^
**SPO2**						
Dex	97.75 ± 1.08	97.53 ± 1.32	97.11 ± 0.95^*^	97.39 ± 1.05	97.17 ± 0.91^*^^#^	97.19 ± 0.95^#^
NS	97.83 ± 0.94	97.53 ± 0.84	97.47 ± 0.94	97.67 ± 1.04	97.67 ± 0.86	97.61 ± 0.73
**NI**						
Dex	98.17 ± 0.85	98.25 ± 0.69	77.00 ± 28.77^*^^#^	35.08 ± 11.73^*^^#^	35.31 ± 5.85^*^^#^	36.08 ± 5.68^*^^#^
NS	98.17 ± 0.74	98.28 ± 0.81	98.31 ± 0.67	94.44 ± 13.39	73.19 ± 29.61^&^	51.67 ± 26.12^&^

*Dex, Intranasal dexmedetomidine group; NS, Intranasal Normal saline group. Compared with the same group at T_0_ time, P < 0.05 versus*

* *Dex*,

& *NS, Compared with NS group at the same time point*,

# *P < 0.05*.

**Figure 3:**
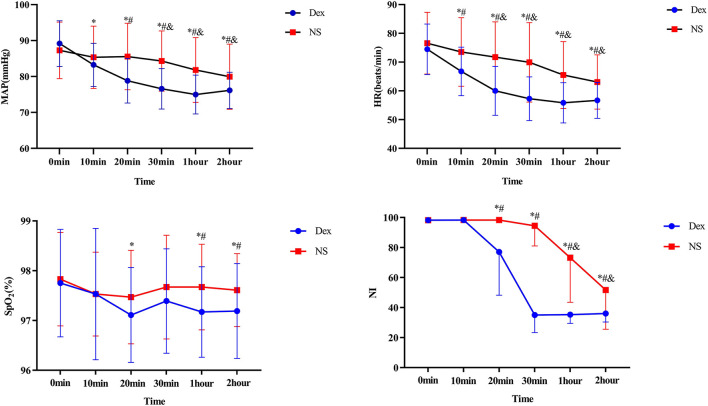
Dex, Intranasal dexmedetomidine group; NS, Intranasal normal saline group. Compared with the same group at T_0_ time, *P* < 0.05 vs. *Dex, ^&^NS. Compared with NS group at the same time point, ^#^*P* < 0.05.

### Adverse Reactions

In the present study, 8 (25%) patients in the DEX group and 4 (12.5%) patients in the NS group had a HR lower than 50 beats/min (*P* = 0.200). Besides, 3 (9.38%) patients in the DEX group and 1 (3.13%) patient in the NS group had systolic blood pressure lower than 90 mmHg. The MAP of these 4 patients was not lower than 30% below baseline, therefore, no medication was used. No respiratory depression was recorded. Drowsiness was observed in 18 (56.25%) patients in the NS group, while no drowsiness was found in the DEX group. No patient felt discomfort after intranasal administration of DEX (Table 4).

**Table 4:** Comparison of other adverse reactions in two groups (cases, %).

	**Respiration depression**	**Hypotension**	**Sinus bradycardia**	**Drowse**	**Nasal discomfort**
Dex	0 (0.00%)	3 (9.38%)	8 (25.00%)	0 (0.00%)	0 (0.00%)
NS	0 (0.00%)	1 (3.13%)	4 (12.50%)	18 (56.25%)	0 (0.00%)
*P*	1.000	0.302	0.200	<0.001	1.000

*Dex, Intranasal dexmedetomidine group; NS, Intranasal Normal saline group*.

## Discussion

It was reported that 38.2% of patients mainly suffered from insomnia due to anxiety on the day before surgery (6). Pre-operative sleep quality has an important influence on post-operative recovery of patients, and poor sleep may increase the incidence of surgical complications (16, 17). Studies have reported the association of insufficient sleep with the increased risk of a variety of human diseases, including heart disease, immune disorders, anxiety, and depression, leading to incurable neurodegenerative diseases (e.g., Alzheimer's disease) (9, 10, 15, 29). Poor pre-operative sleep quality has shown to significantly increase the risk of severe peak pain during exercise after surgery (30). It also increases the incidence of post-operative hyperalgesia (17). Leung et al. (13) revealed the relationship between pre-operative sleep disruption and post-operative delirium. Therefore, improving pre-operative sleep quality may ameliorate a patient's prognosis.

Ellis et al. (31) confirmed that a single session of cognitive behavioral therapy for insomnia (CBT-I) was effective for acute phase. However, patients need to receive treatment for 60–70 min, which limits the use of CBT-I. Benzodiazepine and non-benzodiazepine hypnotics can effectively treat insomnia. However, both have noticeable side effects, including headache, nausea, vomiting, dyspepsia (unpleasant taste), dizziness, drowsiness, migraine, etc. (32). DEX exerts its hypnotic effect by selectively activating presynaptic and postsynaptic central α-2 adrenergic in locus coeruleus. DEX-induced deep sedation mimics stages 2 and 3 of NREM sleep (21, 22).

Intranasal DEX spray may have a better sedative effect than dropping. Xie et al. (33) assessed children's response to peripheral venous intubation after intranasal DEX spray or dropping, and concluded that intranasal DEX spray provided a better sedation. However, there is currently no special dose for DEX atomization in China, and additional atomization devices are required for nasal atomization, causing difficulties in controlling the accuracy of dose, and higher hygienic conditions are therefore required for nasal atomization. The present study aimed to investigate the effects of intranasal DEX administration on sleep quality of patients with pre-operative insomnia, and no spray was herein used. A recent study (34) showed a similar bioavailability (about 40%) for both methods of intranasal delivery (drops or mucosal nebulizer devices). This may be due to the larger mucosal area of the nasal cavity in adults, which has a larger surface area for drug absorption in the nose. The safety and efficacy is not affected by the mode of DEX administration (35). In the future studies, the advantages and disadvantages of these two methods of drug delivery will be further compared.

Expectedly, compared with the NS group, the intranasal DEX improved sleep quality on the night before surgery. Under the standard bodyweight, administration of DEX (2.5 μg per kilogram of bodyweight) could quickly eliminate anxiety and insomnia, and simultaneously improve the total sleep time and sleep quality.

Lirola et al. (36) measured the pharmacokinetics of DEX in adults and found that the onset of clinical sedation was 30–45 min after intranasal administration, in which this onset time was 22.08 ± 3.95 min in the present study, and the difference may be related to the dose of DEX. In Lirola et al.'s study, the dose of intranasal DEX administration was 85 μg, while the dose was based on standard bodyweight in the current study. Miller et al.'s (37) findings also confirmed that the time required for intranasal dextromethorphan administration to reach the lowest effective plasma concentration could be related to the dextromethorphan dose. In Akeju et al.'s (38) study, the total sleep time of patients who received DEX injection was 440.3 min, and the total sleep time of healthy controls was 413.0 min. Compared with our study, the total sleep time of patients who were treated with intravenous DEX was substantially similar to that of patients who were treated with intranasal DEX, while the difference in total sleep time was more significant in the control group. The reason for this difference is that blank control group included patients with pre-operative anxiety and insomnia. The ISI score in the DEX group after treatment was significantly higher than that in the NS group. Besides, no patient in the DEX group felt drowsiness. This is because DEX altered arousal states (38), which is closely related to a natural sleep state, and patients therefore slept with a higher quality.

DEX agonizes α_2_-adrenergic receptors in vascular smooth muscle cells at higher concentrations (39, 40), constricts peripheral blood vessels, and increases blood pressure. In the present study, no hypertension requiring treatment was observed in the DEX group. This could be due to the relatively lower absorption rate of DEX in the nose and the lower maximum plasma concentration, which have been previously confirmed by previous studies. Intranasal dexamethasone is different from the rapid intravenous infusion, and it does not increase BP (37).

Bradycardia is a common complication of DEX, while it typically does not cause serious complications (41). In our study, 8 patients developed bradycardia after receiving intranasal dexamethasone, whereas none reached the intervention level with anticholinergic drugs. No patient had a SPO_2_ below 95% in the study period. Although the blood oxygen at T_2_T_4_ in the DEX group was lower than T_0_ (*P* < 0.05), no actual clinical significance was found.

We asked patients to record any liquid that had flowed into the oral cavity during the nasal administration. One patient in the DEX group took more than 30 min to fall asleep. This indicated that the liquid had flowed into the mouth during the administration period and it was swallowed. A previous study demonstrated that mean absolute bioavailability after peroral, buccal, and intramuscular administration of DEX was 16, 82, and 104%, respectively (42). The failure of this patient to fall asleep within 30 min could be related to a part of the DEX that was swallowed.

## Limitations

Firstly, the sample size of this study is limited, thus, a large-scale multi-center clinical study is required to provide more reliable data to evaluate safety and efficacy of DEX. Secondly, patients' sleep statuses were not assessed using a polysomnography monitor. Third, the dose of DEX required by adults was noticeable, indicating the necessity of multiple intranasal administrations. Changing to a higher concentration of DEX may solve this problem.

## Conclusions

In summary, this study is the first to introduce the potential treatment of DEX in the treatment of pre-operative anxiety insomnia. Intranasal DEX can safely and effectively improve patients' pre-operative anxiety and insomnia. Intranasal DEX may be used as a complementary therapy for insomnia.

## Data Availability Statement

The original contributions presented in the study are included in the article/Supplementary Material, further inquiries can be directed to the corresponding author/s.

## Ethics Statement

The studies involving human participants were reviewed and approved by Scientific Research Ethics Review Committee of the First Affiliated Hospital of Gannan Medical University (LLSC-2020102701). The patients/participants provided their written informed consent to participate in this study.

## Author Contributions

LC and WZ: study design. WZ, XL, XD, KH, MZ, SZ, LZ, and YJ: data collection, analysis, and interpretation. WZ, LC, and YJ: drafting of the manuscript. LC and WL: critical revision of the manuscript. All authors: approval of the final version for publication.

## Funding

This work was supported by the Science and Technology Plan of Health Commission of Jiangxi Province (202130619).

## Conflict of Interest

The authors declare that the research was conducted in the absence of any commercial or financial relationships that could be construed as a potential conflict of interest.

## Publisher's Note

All claims expressed in this article are solely those of the authors and do not necessarily represent those of their affiliated organizations, or those of the publisher, the editors and the reviewers. Any product that may be evaluated in this article, or claim that may be made by its manufacturer, is not guaranteed or endorsed by the publisher.
